# Ingestion of Lycosome L-tug Formulation of Dark Chocolate Ameliorates Postprandial Hyperlipidemia and Hyperglycemia in Healthy Volunteers

**DOI:** 10.1155/2019/1659384

**Published:** 2019-05-16

**Authors:** Ivan M. Petyaev, Natalia E. Chalyk, Victor A. Klochkov, Dmitry V. Pristenskiy, Marina P. Chernyshova, Nigel H. Kyle

**Affiliations:** ^1^Lycotec Ltd, Granta Park, Cambridge, CB21 6GP, UK; ^2^Institute of Cardiology, 12 Chernyshevskogo Str, 410028 Saratov, Russia

## Abstract

Twenty-eight healthy middle-aged volunteers (40-60 years old) with equal gender representation were randomized into 3 study groups to investigate the changes in postprandial glucose and lipids after ingestion of different formulations of dark chocolate (DC). The volunteers from the first group were requested to ingest 100 g of regular DC whereas the individuals from the third group were given 100 g of highly bioavailable lycosome formulated L-tug formulation of DC containing 23.3 mg of lycopene. A second group received a 23.3 mg lycopene capsule, a tomato-derived antioxidant carotenoid as a matching control. Serum specimens were obtained following 30 minutes as well as 1, 2, and 3 hours after study products intake. Ingestion of L-tug DC was accompanied by the reduced postprandial hyperglycemia with maximum difference seen at 3^rd^ hour of the study and reduction of average AUC_Gluc_ values by 20% (P<0.05) as compared to regular DC. Moreover, ingestion of L-tug DC was accompanied by a statistically significantly reduced median concentration for postprandial triglycerides (to 390.7 mg⁎hr/dL; 5/95%% CIs: 363.2/405.7 versus regular DC value of 439.5mg⁎hr/dL and a lower range of confidence intervals - 5/95%CIs: 394.0/475.1). A similar tendency was observed in changes of total cholesterol concentration. Ingestion of L-tug DC completely abolished total cholesterol increase seen in volunteers at 3^rd^ hour of postprandial period following intake of the control DC. Ingestion of lycopene alone did not cause any changes in postprandial changes of glucose or serum lipids. The observed postprandial changes can be related to the 56.2 % increase in serum lycopene level which was observed after ingestion of L-tug DC only. Higher serum lycopene levels following the ingestion of L-tug DC resulted in a corresponding increase in serum antioxidant capacity and reduction of oxidized LDL as well as a decline in malonic dialdehyde concentration in the serum of volunteers.

## 1. Introduction

The medicinal properties of dark chocolate (DC) and other foodstuffs derived from* Theobroma cacao* were first noted centuries ago dating back to the time of Christopher Columbus whose expeditions first brought cocoa to Europe and introduced DC into European culinary culture [1, 2]. Amelioration of fatigue, indigestion, and other gastrointestinal disorders were attributed to the potential medicinal benefits associated with regular consumption of DC [1]. Current nutritional science and numerous* in vitro* and* in vivo* studies suggest that DC intake may have a measurable effect on cardiovascular health, the central nervous system, and intermediate metabolism [3]. DC contains nearly 300 identifiable chemical substances with conceivable biological activity. However, the medicinal effects of DC seem to be mediated largely by cocoa polyphenols, methylxanthines (caffeine, theobromine), and certain minerals: magnesium, zinc, and iron [1, 4]. The molecular interplay of these biologically active compounds and their individual role in the effects of DC are yet to be fully explored. However, it has been demonstrated that some of the physiological changes attributed to DC ingestion occur with the participation of a nitric oxide-mediated pathway, resulting in nitric oxide production* via* Ca^2+^-independent activation/phosphorylation of eNOS [3, 5]. Yet, there are legitimate concerns related to the consumption of DC by individuals with compromised health status. Currently, there are no dietary guidelines and/or clear recommendations regarding daily intake of DC. Confectionery brands of DC contain more than 50g of fat, 20g of carbohydrates, half of which is sugar, with a total of 600 calories per 100 g [1]. Consequently, regular consumption of large amounts of DC may affect the outcomes of hyperlipidemia and type 2 diabetes mellitus. Therefore, there is a need to develop a new nutraceutical formulation of DC with limited impact on lipid and carbohydrate homeostasis.

As we reported recently [6, 7], lycosome formulation of DC containing carotenoids embedded into DC matrix (L-tug DC) has a number of beneficial features including greater bioavailability of cocoa polyphenols and the ability to reduce the concentration of oxidized low density lipoproteins (ox-LDL) as well as other markers of biological oxidation and systemic blood pressure. In the present study we evaluate the effect of L-tug DC on postprandial lipids and glucose levels following acute ingestion as this had not been addressed previously.

## 2. Material and Methods

### 2.1. Study Objectives

The study was conducted by Lycotec Ltd (Cambridge, UK) and the Institute of Cardiology (Saratov State University, Russian Federation). The study was conducted as part of larger clinical trial under a protocol approved by the local Ethical Committee and registered (ACTRN12613000966796). The study was designed as a pilot, double-blind, interventional, randomized clinical study involving healthy middle-aged volunteers.

The main objective of the study was to compare postprandial changes in serum lipids and glucose levels after single ingestion of 100 g of L-tug DC in comparison with the same amount of control DC over the first three-hour time window. The secondary objectives of the study included assessment of postprandial changes in markers of inflammatory and oxidative damage, which appear in the blood as a response to the transient challenge to the liver by postprandial lipids. In addition, total antioxidant capacity of the serum and serum lycopene levels were measured following ingestion of L-tug DC and DC. Since the former contained embedded lycopene, a separate group of volunteers was assigned to ingest the same dose of lycopene in capsule form without the intake of DC.

All volunteers were aware of the purpose of the study and signed a written consent form regarding their participation. All volunteers underwent physical and laboratory examination and their medical history was evaluated during the enrolment period.

### 2.2. Subjects and Inclusion/Exclusion Criteria

Healthy Caucasian male and female subjects 40–60 years old were enrolled in the study. Major inclusion criteria were assessed at the screening visit and were as follows: Caucasian individuals of both genders, aged 40-60 years, absence of any current disease, normal vital signs (blood pressure, body temperature, pulse, and respiration rate), and normal body mass index (BMI 18.5-24.9), as well as normal blood cell count and liver function parameters, no current intake of lipid-lowering, or any antidiabetic drugs. The exclusion criteria were inability to comply with the study protocol, concurrent medical conditions (hepatitis, pancreatitis, diabetes, cancer, recent cardiovascular events, tuberculosis, etc.), smoking (> 10 cigarettes per day), and intolerance to cocoa and tomato products.

### 2.3. Study Products

#### 2.3.1. Dark Chocolate

Dark chocolate bars (100 g) with 85% cocoa from Green & Black's Organic (Uxbridge, UK) were used in the study. Nutritional characteristics per 100 g were as follows: calories - 630 kcal; protein – 9.2 g; fat – 53.5 g; and carbohydrates – 22.2 g, of which sugar was 13.8 g. The chocolate was melted, treated, and tempered in exactly the same way for all groups of the study regardless of the addition of lycopene.

#### 2.3.2. L-tug™ Formulation of Dark Chocolate

Modification of cocoa butter triglycerides by lycopene embedment into DC is accompanied by changes in the DC crystallization [6, 7]. This enhances bioavailability of cocoa flavanol compounds by protecting them from oxidative modification during the digestive process [6, 7]. The concentration of lycopene in the L-tug chocolate was 23.3 mg per 100 g bar. The lycopene used was in the form of tomato oleoresin from Lycored Inc. (New Jersey, NJ, USA) and contained 97% trans-isomers and 3% cis-isomers.

#### 2.3.3. Lycopene

Lycopene control capsules containing 23.3 mg of lycopene were made from the same batch of tomato oleoresin that was used for the preparation of the L-tug DC.

### 2.4. Study Protocol

The volunteers were asked to refrain from consumption of cocoa- and tomato-based products for 10 days before beginning the study. The study participants were also asked to make no changes to their diet and lifestyle and to avoid vigorous physical activity and excessive alcohol consumption before the test procedure.

The volunteers were asked to arrive at the study site following overnight fasting (10-12 hours) and blood specimens were obtained within 30 minutes of arrival. The test products (100 g of DC with or without lycopene or 23.3 mg lycopene capsule) were given at the “0” time point of the study after obtaining fasting blood specimens. Volunteers were asked to consume the products within 5 minutes. From this time point a 3-hour observational period began. Volunteers were asked to remain in a sitting position and were allowed to consume 250 ml of regular drinking water with no food served before or after ingestion of the study products. Blood sampling was conducted at the “0” time point as well as after 30, 60, 120, and 180 minutes following consumption of study products. Neither the volunteers nor the study personnel was informed of the specification of the dark chocolate bars (presence or absence of lycopene in the DC matrix) during the interventional period or serum specimen processing/analysis. The lycopene alone ingestion group was unblinded for both the study personnel and the volunteers. An independent statistician and the principal investigator conducted the decoding of the results. All volunteers and study personnel were informed of the study aims and objectives and had signed written consent for participation in the study.

### 2.5. Blood Sampling

Blood specimens were taken at the time points indicated above by qualified personnel. The specimens were allowed to clot at 37°C for 30 minutes and were then centrifuged immediately in a refrigerated centrifuge (4°C) at 3000 rpm for 10 minutes. The serum was separated and aliquots dispensed prior freezing at -80°C. All quantification assays were performed at the same time in side-by-side assays with identical standards and reagents.

### 2.6. Biochemical Analysis

Total cholesterol (TC), triglycerides (TAG), HDL/LDL cholesterol, glucose, C-reactive protein (CRP), and alanine/aspartate aminotransferases (AST/ALT) were measured using a Biosystem A25 automated analyzer (Applied Biosystems, Grand Island, NY) using BioSys kits and calibrators.

Total antioxidant capacity (TAC): frozen serum specimens were analyzed within 10 days of collection using Biorex reagents according to the manufacturer's instructions (Biorex Diagnostics, Antrim, UK). Results were expressed as mmol of Trolox equivalent (TE) per liter (mM TE/L).

Oxidized LDL and malonic dialdehyde values were measured in serum specimens as previously described in our work [5, 6].

Serum lycopene analysis was performed as recently described [8]. Briefly, 400 *μ*l of serum was mixed with 400 *μ*l of ethanol and was extracted twice with 2 ml hexane. The combined hexane layers were evaporated to dryness in a vacuum (Scan Speed 32 centrifuge) and the residue reconstituted to a volume of 100 *μ*l in sample solution (absolute ethanol—methylene chloride, 5:1, v/v). The specimens were centrifuged again (15 min at 10,000* g*) and clear supernatant was transferred to HPLC vials. Five microliters of the extract was injected into an Acquity HSS T3 75 x 2.1 mm 1.8 *μ*m column (Waters, MA, USA) preceded by an Acquity HSS T3 1.8 *μ*m VanGuard precolumn (Waters) and eluted isocratically at 45°C with the mobile phase (acetonitrile—0.08% phosphoric acid solution—tert‐butyl methyl ether, 70:5:25, v/v/v) at a flow rate of 0.5 ml/min. The lycopene peak was detected by a photodiode array detector (Waters) at 474 nm. The peak area was measured using Empower 3 software (Waters). The lycopene concentration in serum samples was calculated by reference to an analytical standard (lycopene from tomato, L9879; Sigma, USA).

### 2.7. Statistical Analysis

The results are shown as averages with standard deviation. For the assessment of normally distributed parameters, the Shapiro–Wilk method was used. Student's t-test was then applied for both paired and unpaired samples. Between-group differences at one time point were evaluated by the Wilcoxon–Mann–Whitney test (continuous variables) and Fisher's exact test (categorical variables). Serum levels of glucose and TAG were analyzed additionally using median values and 5% and 95% confidence intervals with box and whisker plot analysis. The 3-hour area under curve (AUC) was calculated using one-way ANOVA with further analysis using Tukey HSD test. Data analysis was performed using Stata SE (College Station, TX), version 12.1. All statistical tests were two-sided and statistical significance level alpha was set at 0.05 for the analysis.

## 3. Results

### 3.1. Baseline Parameters

Table 1 shows the variations of major baseline values in the volunteers enrolled in the study. As can be seen from Table 1, all participants enrolled in the study had normal BMI values and were normoglycemic and normolipidemic under conditions of overnight fasting. Moreover, normal serum AST and ALT levels suggest uncompromised liver function. No statistically significant variations were seen in gender representation and other baseline physiological values among the three groups of volunteers enrolled in the study.

### 3.2. Glucose Concentration

**Figure 1:**
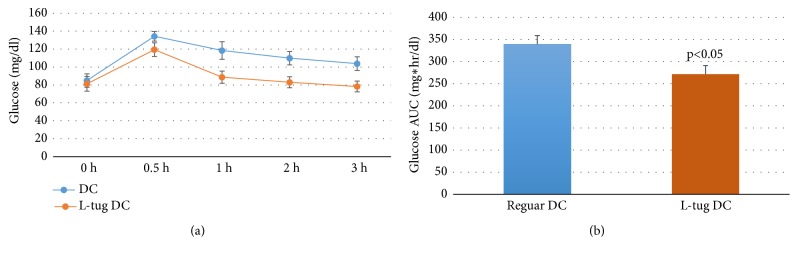
Changes in serum glucose (a) and area under the curve (AUC, (b)) for serum glucose concentration after ingestion of regular and L-tug dark chocolate (DC).

Figure 1 shows the dynamics of changes in serum glucose level after ingestion of control and L-tug dark chocolate. Both DC formulations gave a modest but statistically significant increase (p<0.05) in serum glucose values over the postabsorption period with a maximum peak registered 30 minutes after DC intake. However, the serum glucose levels were noticeably higher following ingestion of the regular formulation of dark chocolate at all-time points studied (Figure 1(a)) which was reflected by a higher mean AUC value as compared to L-tug DC (Figure 1(b)). In particular, ingestion of regular DC gave an increase in average serum glucose concentration by 52.2, 34.0, 23.8, and 17.0 % after 30 minutes or 1, 2, and 3 hours, respectively, of the postingestion period, whereas the corresponding increases in serum glucose after L-tug DC ingestion were distinctly less significant (40.2; 9.1; 2.09; and -3.6% accordingly). This muted glycemic response resulted in lower AUC values (Figure 1(b)) in the L-tug DC group (reduction of average AUC values by 20.0%).

Ingestion of lycopene alone in capsule form did not give any measurable changes in serum glucose levels (results not plotted).

### 3.3. Triglyceride Levels

**Figure 2:**
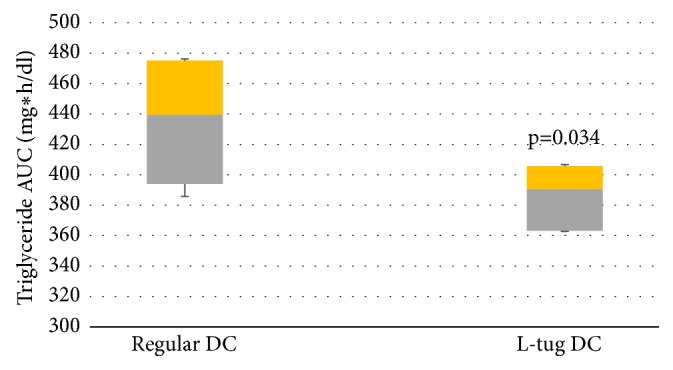
Changes in area under the curve (AUC) values for serum triglyceride concentration after ingestion of regular and L-tug DC.

Regardless of the study group, an increase in average TAG values in serum took place over the course of 1 hour following DC ingestion with no statistically significant differences at the 30-minute time point or at the first hour following ingestion of DC for either group. However, after the second hour and especially after the third hour of the observational period the serum triglyceride level was higher in volunteers ingesting regular DC (p<0.05). This resulted in reduction of TAG_AUC_ shown in Figure 2.

The ingestion of L-tug DC was accompanied by a lower median for AUC_TAG_ (390.7 mg*∗*hr/dl) and a lower range of confidence intervals (5/95%CIs: 394.0/475.1)) as compared to regular formulation of DC (median - 439.5mg*∗*hr/dl; 5/95% CIs: 363.2/405.7). This difference was statistically significant with a* p* value of 0.034.

The analysis of statistical differences in paired variables (“0” time point versus third hour of the postprandial period) revealed that ingestion of L-tug DC leads to a statistically significant reduction in medians reflecting serum TAG concentration as compared to regular DC. The net increase in the postprandial serum TAG content after ingestion of regular DC was +18.0 mg/dl (5/95% CIs:4.8/31.2) whereas the value for L-tug DC was +5.0 mg/dl (5/95% CIs: 1.4/8.6, p<0.05) suggesting altogether a 2.6-fold difference between the groups.

Once again, ingestion of lycopene alone did not result in any significant changes to triglyceride concentration at any of the time points studied (results not plotted).

### 3.4. Total Cholesterol (TC)

**Figure 3:**
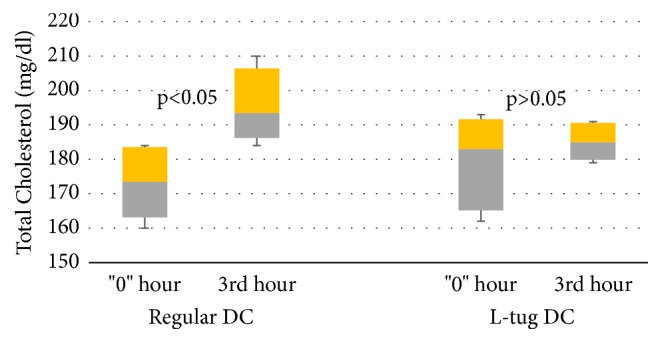
Changes in serum total cholesterol concentration after ingestion of regular and L-tug DC. (“p” reflects the statistical difference between initial and final values).

Changes in TC concentration developed in a manner similar to the triglyceride dynamics described above. The variations in TC content were statistically insignificant at early time points of the observational period. However, at the third hour of the postprandial period there was a noticeable difference between the groups (Figure 3). There was an increase in TC concentration for the regular DC group in both medians (from 173.5 mg/dl to 193.5 mg/dl) as well as confidence intervals (from 5/95% CIs: 163.1/183.5 to 186.2/206.4, p<0.05). On the other hand, there were no changes in serum TC content for the L-tug DC group at the third hour of the postprandial period (p>0.05). Ingestion of lycopene alone did not result in changes to TC concentrations (results not plotted).

### 3.5. Serum Lycopene Values

**Figure 4:**
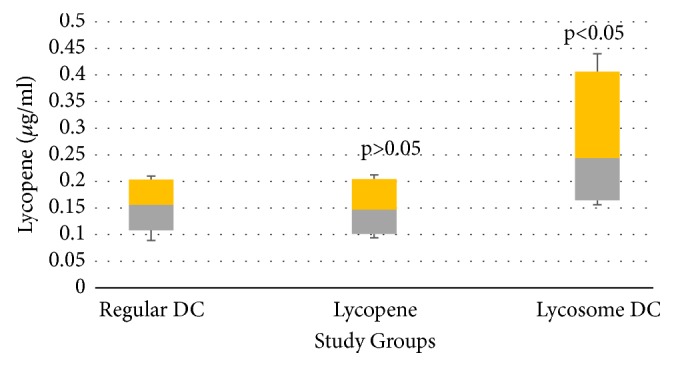
Box-and-whisker analysis. Serum lycopene level after 3 hours following ingestion of L-tug DC (“*p*” value reflects statistical difference in lycopene concentration among study groups as compared to regular DC group).

As can be seen from Table 2, ingestion of regular DC did not affect postprandial value of serum lycopene concentration at the end point of the observational period. No immediate effect of ingestion of lycopene alone was seen on serum lycopene level in the 3-hour time window following ingestion either. However, there was a statistically significant increase in serum lycopene concentration (Table 2) in volunteers ingesting L-tug DC at the end of the third hour following intake.  Figure 4 provides a better insight into variations of lycopene concentration at the end point of the observational period in the study groups. Serum levels of lycopene were almost equal in the volunteers ingesting either regular DC or lycopene alone (median values of 0.156 and 0.147 *μ*g/ml, respectively). However, ingestion of L-tug DC resulted in much higher values for lycopene concentration at the end of the observational period (median - 0.244 *μ*g/ml, 5/95% CIs: 0.164/0.406).

### 3.6. Serum Total Antioxidant Capacity (TAC)

**Figure 5:**
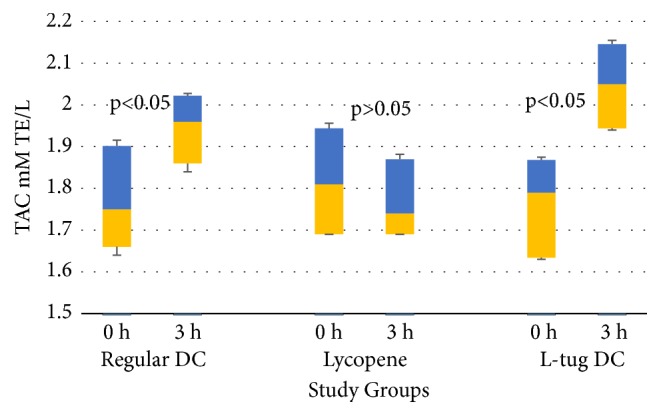
Box-and-whisker analysis. Postprandial changes in total antioxidant capacity of serum in volunteers following ingestion of L-tug DC. “*p*” values reflect intragroup differences between initial and final values.

There was a different pattern of changes for TAC parameters at the end point of the observational period. As can be seen from Figure 5, ingestion of regular DC was accompanied by a small postprandial increase in TAC medians-up to 1.96 mM TE/L (5/95% Cis: 1.86/2.02) as compared to time “0” (1.75 mM TE/L, 5/95% Cis:1.66/0.40). Ingestion of lycopene alone did not give any changes in TAC (p>0.05), which is consistent with the unchanged serum lycopene level for this group shown in Table 2. However, the most significant increase in TAC values took place in the serum of volunteers who ingested L-tug DC (2.05 mM TE/L; 5/95% Cis: 1.94/2.14) versus time “0” – median 1.74 mM TE/L (5/95% Cis:1.61/1.86).

### 3.7. Oxidized LDL (ox-LDL) and Malonic Dialdehyde (MDA)

Table 3 shows between group differences in ox-LDL and malonic dialdehyde concentrations at the end of the observational period. As can be seen, volunteers ingesting regular DC or lycopene alone showed no statistically significant difference in ox-LDL or MDA concentration. However, there was a postprandial reduction in average ox-LDL and MDA levels following the ingestion of L-tug DC (by 22.7% and 36.8 %, respectively).

## 4. Discussion

Postprandial increases in serum triglyceride, cholesterol, and glucose levels are a normal physiological response to fat and carbohydrate containing meals which depend on health status, age, composition of meals, and time following intake. Recently these increases have gained particular attention as new discoveries reveal their key significance for human health. Repeated episodes of postprandial hyperglycemia (>140 mg/dl) are associated with a two-fold increase in risk of death from cardiovascular disease in healthy normoglycemic individuals with HB1c <6.1 [9, 10]. Similarly, increased postprandial TAG responses are now considered as an independent risk factor for atherosclerosis. An increase of 1 mmol/l in fasting TAG level increases risk of the cardiovascular disease by 14% [11].

As shown, ingestion of L-tug formulation of DC blunts postprandial increases in triglycerides, cholesterol, and serum glucose as compared to control DC without lycopene.

The decline in postprandial lipids observed in volunteers ingesting L-tug DC is consistent with our previously reported observation that ingestion of L-tug DC resulted in reduction of the fasting level of these lipids [6]. Incorporation of lycopene, a highly lipophilic compound, into cocoa butter triglycerides enlarges the size of their globular / crystal structures in the chocolate matrix which results in reduction of their digestibility. In addition, lycosome particles are relatively stable in the gastrointestinal environment and are known to penetrate the intestinal epithelium at least partially in native form [12]. Presence of lycopene, a powerful ligand for hepatic carotenoid receptors, in the outer layer of lycosome particles may promote faster and more efficient clearance from the bloodstream via hepatic scavenger receptors and ameliorate thereby postprandial hypertriglyceridemia and hypercholesterolemia.

Serum lycopene changes observed in our study and possible relation to postprandial responses require careful consideration. Lycopene is highly hydrophobic and poorly absorbed from the gastrointestinal lumen [13, 14]. Even long-term consumption of lycopene-containing foodstuffs and / or lycopene supplements may not lead to significant increase in serum concentration, especially in elderly individuals [14]. Thus, the unchanged lycopene levels seen in volunteers ingesting lycopene alone correlate well with reports revealing low bioavailability of lycopene [13–15]. Coingestion of lycopene with fat-containing products promotes its intestinal absorption [16], explaining the increase in lycopene level with L-tug DC.

A combination of two possible mechanisms, reduction in digestibility of cocoa butter, and formation of lycosome structures, which provide increased bioavailability for both lycopene and cocoa bioactives [17], seems the most likely explanation for the beneficial effects of L-tug DC.

It was surprising and encouraging to observe a reduction in postprandial hyperglycemia following ingestion of L-tug DC. There is no well-documented link between dietary lycopene and frequency of type 2 diabetes mellitus [18]. However, recent results show that lycopene may increase insulin sensitivity and prevent hyperglycemia through inhibition of the STAT-3/SREBP-1c pathway [19]. Moreover, cocoa flavanols seem to affect glucose turnover, reportedly enhancing insulin secretion and sensitivity reducing thereby glucose levels [20]. Therefore, potentiated effect of lycopene and cocoa polyphenols on glucose hemostasis may reduce postprandial hyperglycemia as observed with L-tug DC. This is in good agreement with changes in total antioxidant capacity (TAC), which mirrored increase in serum lycopene concentration. As shown above, ingestion of regular DC and especially L-tug DC gives statistically significant postprandial increases in TAC values accompanied in the last group of the study by reduction in postprandial concentration of ox-LDL and malonic dialdehyde. This reveals that postprandial increase in serum lycopene content following the ingestion of L-tug DC is essential for amelioration of oxidative stress markers even in healthy individuals. Elucidation of a potential link between serum antioxidant levels, postprandial hyperglycemia, and markers of oxidative stress requires further thorough investigation.

Our study has some limitations: ingestion of 100g of DC is not a recommendable dietary option and was only used for testing purposes. Future studies should verify if smaller amounts of DC with higher amounts of lycopene would help maintain glucose and TAG levels, especially in subjects with metabolic syndrome and type 2 diabetes patients. A higher number of volunteers would obviously be required for further evaluation.

## Figures and Tables

**Table 1:** *Baseline values of fasting study participants* (averages±SD).

Parameter	Study Groups		
	Regular DC	Lycopene	L-tug DC
Age	49.3±4.1	54.3±3.7	51.6±5.3

Men/Women	4/5	4/4	5/5

BMI	22.3±1.1	21.5±1.4	23.0±1.2

Serum Glucose mg/dl	85.3±5.6	81.3±6.3	79.4±4.9

Serum Triglycerides mg/dl	130.6±7.3	121.4±4.2	129.9±5.3

Total Cholesterol mg/dl	167.5±8.3	174.6±14.5	181.5±12.3

LDL Cholesterol mg/dl	112.8±8.3	127.5±12.5	105.6±10.3

HDL Cholesterol mg/dl	37.2±3.1	41.4±2.9	39.4±3.3

AST IU/ml	34.3±6.3	27.2±7.4	35.9±4.2

ALT IU/ml	44.6±3.5	37.8±6.9	45.6±5.5

CRP mg/l	2.1±0.6	1.7±0.8	2.3±0.4

TAC	1.76±0.09	1.79±0.09	1.74±0.10

Pulse Rate beats/min	74.3±4.22	71.8±3.99	76.5±4.76

Blood Pressure mm Hg			
Systolic:	108.3±6.2	110.1±4.1	111.7±4.5
Diastolic:	78.3±5.3	75.3±5.8	74.6±4.1

**Table 2:** Serum lycopene concentration (*μ*g/ml) in volunteers at 3^rd⁡^ hour after ingestion L-tug DC (averages±SD).

Time Point	Study Groups		
	Regular DC	Lycopene	L-tug DC
“0” hour	0.14±0.04	0.13±0.04	0.15±0.03

“3”hour	0.16±0.03	0.15±0.03	0.25±0.07*∗*

(*∗*) – p<0.05

**Table 3:** Serum oxidized LDL (ox-LDL) and malonic dialdehyde (MDA) levels in volunteers 3 hours after ingestion of dc formulations (averages±SD).

Study Groups	“0” Hour	“3^rd^” hour
Ox-LDL (ELISA UNITS x 10^−3^)		

Regular DC	631.58±16.21	611.56±14.11

Lycopene	644.65±12.95	669.43±15.33

L-tug DC	674.78±12.32	521.23±18.21^*∗*^

MDA (*μ*M)		

Regular DC	123.67±8.31	141.21±11.21

Lycopene	131.25±9.94	118.25±10.41

L-tug DC	119.25±8.66	75.34±12.33^*∗*^

(*∗*) – p<0.05

## Data Availability

The supporting results will be displayed on the publicly available website Lycotec.com. Moreover, the data that support the findings of this study are available from the corresponding author, Dr. Ivan M Petyaev, upon reasonable request.
